# Determinants of participation in glaucoma genomic research in South East Nigeria: A cross-sectional analytical study

**DOI:** 10.1371/journal.pone.0289643

**Published:** 2023-11-17

**Authors:** Nkechinyere J. Uche, Onochie Okoye, Nkiru Kizor-Akaraiwe, Chimdi Chuka-Okosa, Enoch O. Uche

**Affiliations:** 1 Department of Ophthalmology, University of Nigeria Teaching Hospital, Ituku Ozalla, Enugu, Nigeria; 2 Department of Ophthalmology, ESUT Teaching Hospital Parklane, Enugu, Nigeria; 3 Department of Surgery, University of Nigeria Teaching Hospital, Ituku Ozalla, Enugu, Nigeria; Johns Hopkins University School of Medicine, UNITED STATES

## Abstract

**Background:**

Genomic research advances **the** understanding of human health and disease. It also drives both the discovery of salient genetic association(s) as well as targeted screening, diagnostic and therapeutic strategies. Human subject participation is crucial for the success of genomic research.

**Methods:**

This is a cross sectional analytical study conducted at two tertiary centers in Enugu Southeast Nigeria. Semi structured questionnaires were administered to eligible consenting participants. Data on their demographics, willingness to participate in genomic research and motivation for participation were obtained. Data was analyzed using Stata version 17 and summarized using median, frequencies and interquartile range(IQR). Associations between covariates were evaluated with Chi square test and multivariable logistic regression.

**Results:**

Among 228 glaucoma subjects who participated in our study,119(52.2%) were female and 109(47.8%) were male. The median age was 64 years(IQR = 50–76). Although 219 (96.0%) participants expressed willingness to participate in a glaucoma genetic study, only 27(11.9%) of them will be willing to participate if there will not be feedback of results to participants (χ^2^ = 18.59, P<0.001). No participant expressed willingness to submit ocular tissue samples. Majority (96.2%) of subjects will not participate if the intended research required submission of body samples after death. Desire to know more about glaucoma (63%) was the most common reason for participation. In a multivariable logistic model, subjects between 61–90 years (p = 0.004, OR = 7.2) were 7 times more likely to express willingness to participate in glaucoma genetic research after adjusting for other covariates when compared to subjects aged 41–60 years. Other covariates did not influence participants’ willingness.

**Conclusion:**

Glaucoma subjects are more likely to be willing to participate in genetic research, if they would receive feedback of results. Willingness to participate in genetic research is significantly associated with age.

**Limitations:**

We did not evaluate the salient options for feedback of results to participants in our study.

## Background

Genomic research has broadly advanced the understanding of human health and disease. It has also catalyzed the development of strategies that apply scientific knowledge obtained from the discovery of salient genetic association(s) in the targeted screening, diagnosis and therapy of diverse health disorders such as glaucoma [1, 2]. Glaucoma is a group of genetically complex disorders characterized by progressive degeneration of the optic nerve, loss of retinal ganglion cells, thinning of retinal nerve fiber layer, and increasing excavation of the optic disc, accompanied by characteristic visual field changes [3]. Elevated intraocular pressure, older age, a positive family history of glaucoma are identified risk factors [4, 5]. Glaucoma is the leading cause of irreversible blindness worldwide [6]. Males have a higher primary open angle glaucoma burden than females [5, 7]. It is estimated globally that about 111.8 million persons 40 -80years will have glaucoma by 2040 with Africa accounting for 17% [8].

According to the Nigeria National Blindness and Visual impairment Survey, the prevalence of glaucoma is 5.02% for all glaucoma types in persons 40 years and above [9] with 1 in 5 persons with glaucoma already blind from the disease [10]. Primary open angle glaucoma [POAG] is the most common subtype of glaucoma in Nigeria [9]. Within Nigeria, the Ibos in the southeast region have the highest prevalence [6.1%] of primary open angle glaucoma compared to other regions [5].

The prevalence of glaucoma in Nigeria appears higher than the reported rate from South Africa [4.5%] [11], Tanzania [2.6%] [12] among the Chinese population as reported in the Singapore Chinese eye study [4.0%] [13], as well as Europe [2.8%] [14]. At 3.54%, the global prevalence of glaucoma is lower than the reported rates from Africa and Asia [8].

Although subtypes of glaucoma are reported to be prevalent and more clinically severe among populations of African ancestry [15, 16], there are few genomic studies reporting on glaucoma patients from African populations [15]. Some genome wide association studies (GWAS) have identified several single nucleotide polymorphisms (SNPs) which are linked to POAG [17, 18], however these SNPs have been found to play little or no role in populations of African ancestry [18]. However, other GWAS have suggested a possible causal linkage between the *APBB2* locus (amyloid–β A4 precursor protein-binding family B member 2: APBB2; chromosome 4, rs59892895T>C) and POAG specifically among persons of African ancestry including Nigeria [15, 19].

As glaucoma genomic research continues to expand in scope and application, human subject participation remains a critical resource because the analysis of ethically obtained biological samples from consenting human participants is an obligatory requirement for the successful conduct of a well-designed genomic glaucoma study aimed at providing a valid scientific evidence. However, certain factors [socio-demographic and racial] may determine participant’s willingness to enroll in genomic research [20].

To date there are no studies from our sub region that provide scientific data on the factors that determine participation of glaucoma patients in genomic research. Our study is therefore aimed at bridging this gap as well as providing the foundational evidence for further scientific inquiry.

## Materials and methods

We conducted a cross sectional analytical study of consecutive participants attending the eye clinics at the ophthalmology units of the University of Nigeria Teaching Hospital and the Enugu State University Teaching Hospital Parklane Enugu between April 2022 and January 2023. Both tertiary hospitals are located in Enugu state southeast Nigeria and boast of well-established glaucoma units that apply similar care protocols. Both centers are study sites in the Eyes of Africa: The Genetics of blindness research. Semi structured questionnaires were administered to eligible consenting participants by the research assistants through a face to face interview, after the delivery of relevant information to them using the participant information sheet.

The participant information sheet was administered to educate the participants on glaucoma genomic research in a language they could easily understand.

All study participants received information about glaucoma genomic research. Specifically, they were informed that the genetic code of humans was transmissible from parents to their offspring, hence, heritable illnesses could be transmitted through the genetic code from one’s parents. They were also informed that the genetic code could be elucidated by testing samples obtained from the human body using special diagnostic techniques. As a result, genetically transmissible diseases such as glaucoma can be identified by examining human samples obtained from individuals. Further, while testing for genes that cause genetically inherited diseases such as glaucoma, it is possible to also discover genes that may be responsible for the causation of some other diseases as well as obtain health information that may apply to participant’s family members as well. In addition, glaucoma genomic research may identify the specific causal genetic factors responsible for glaucoma subtypes prevalent in our environment.

Following this process, the semi structured questionnaire was then administered both in English and in the participants local language.

### Inclusion criteria

Consenting participants who are 40 years and above with the under listed characteristics:

Diagnosis of Primary Open Angle Glaucoma(POAG)

Eligibility for enrolment into the Eyes of Africa genetics of blindness study.

### Exclusion criteria

Participants excluded from the study had the following characteristics

Previous participation in glaucoma genetic research

Non consenting POAG subjects

Less than 40 years of age

Clinical subtypes of glaucoma other than POAG

### Diagnostic criteria

Diagnosis of primary open angle glaucoma was based on the following findings: a glaucomatous optic neuropathy with corresponding characteristic visual field changes, open angles on gonioscopy and intraocular pressure more than 21mmHg, in the absence of any other secondary cause of optic neuropathy.

### Sample size estimation

We estimated the sample size with the formula (Z_1-à/2_)^2^ p(1-p)/d^2^. Z_1-à/2_ = Standard normal variate at 5% type 1 error rate(P<0.05), p = prevalence of willingness to participate in a previous genetic study (85%) [21], d = precision (5%) (Z_1-à/2_) = 1.96, Prevalence of willingness = 85%(0.85), 1-p = 0.15, d = 5. Hence (Z_1-à/2_)^2^ p(1-p)/d^2^ = 1.96 x 1.96 x 85 x 15/5 x5 = 195.9.

This sample size will give us 80% power to detect at least 82.5% (85-d/2) prevalence of willingness to participate in a genetic research from our study sample. To account for withdrawal of consent as well as incomplete records and missing data, we added 20% to this estimate. Our new sample size = 195.9 + 20/100 x 195 = 235.0. Within these limits, we believe that we are able to exclude incomplete records and the few cases of missing data without compromise of statistical power.

### Study variables

Our primary outcome(dependent) variable is willingness to participate in glaucoma genetic research. The exposure (predictor) variables are socio-demographic factors age, gender, marital status, education, employment and family history of glaucoma. We suspect that any association with age and willingness to participate in glaucoma genetic research may be confounded by education, gender, marital status, employment and family history. We further suspect that any of the following socio-demographic and clinical factors, education, family history of glaucoma, employment, marital status and gender could serve as effect modifiers as well.

### Data handling and analysis

Data was collected on participants age, gender, educational status, marital status, employment status willingness to participate in genomic research, willingness to submit blood samples and ocular tissue sample, willingness to submit body and ocular tissue after death and motivations for participation using a semi-structured questionnaire. Prior to study commencement, to achieve a high interviewer reliability and reduce bias, two research assistants were trained to administer the questionnaire to subjects using face to face interviewer method. Data was captured using Excel spreadsheet and the data from excel was imported into Stata version 17 and analyzed. The data was summarized using median, frequencies and interquartile range. Associations between covariates were evaluated with Pearson’s χ^2^ test. Bivariate(crude) analysis and multivariate (adjusted) analysis were performed with multivariable logistic regression to evaluate the effect sizes and directionality of associations between covariates and willingness to participate in glaucoma genetic research. We used a multivariable model to adjust for possible confounders. We also generated interaction terms to evaluate for effect modification. Inferences on statistical associations were made using the 95% level and a *P* value <0.05 was considered statistically significant.

### Outcome variables and covariates

The primary outcome(dependent) variable for our study is the willingness to participate in glaucoma genomic research, while the covariates are subjects socio-demographic variables.

### Ethical approval

Approval and permission to conduct this study were obtained from both the Health Research Ethics Committee of UNTH and ESUTH respectively. Written informed consent was obtained from all study participants. Authors did not have access to information that could identify or unmask individual participants’ identities in any form during and after data collection.

## Results: Socio-demographic factors

**Fig 1:**
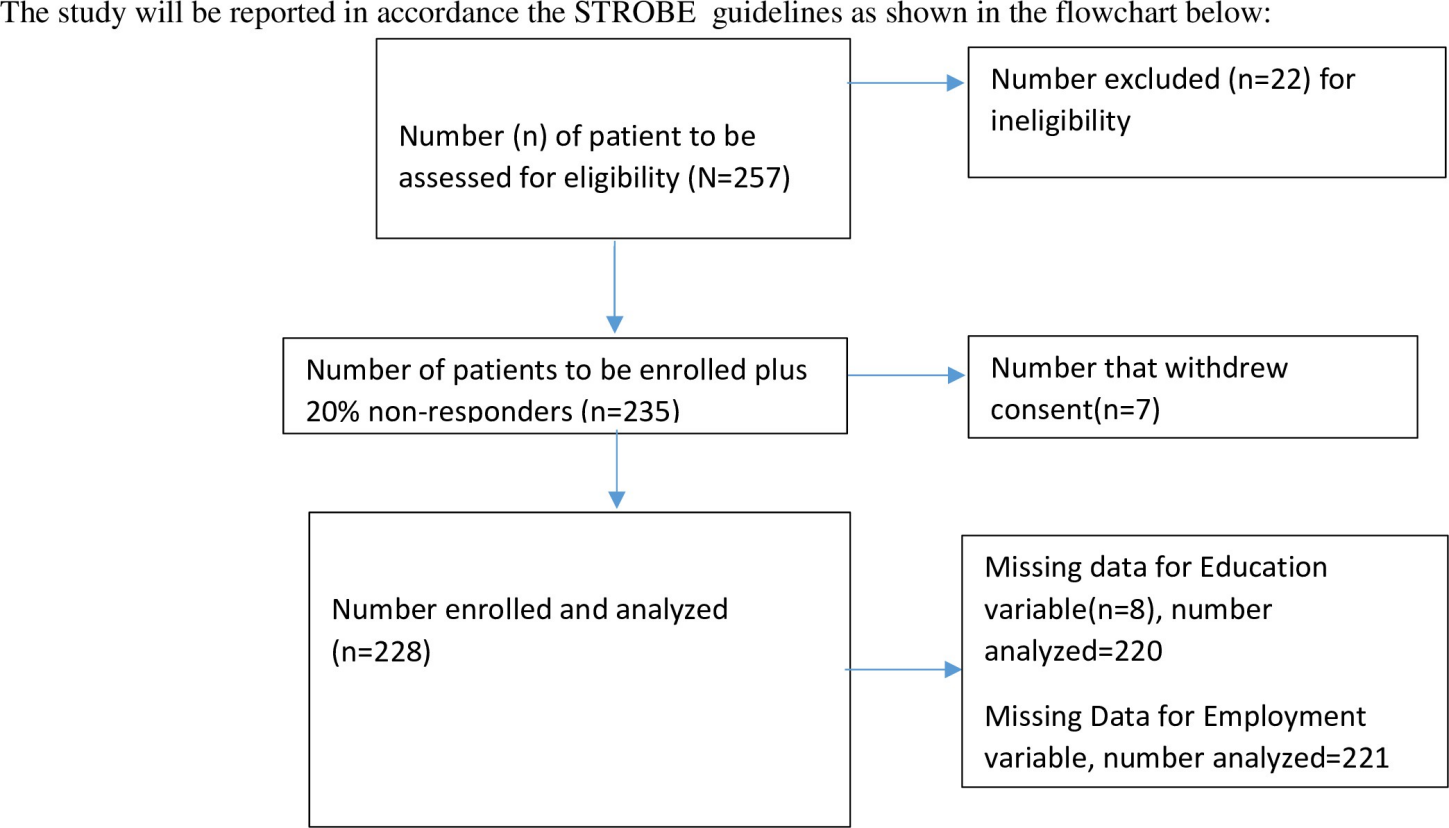
Study flow chart.

**Fig 2:**
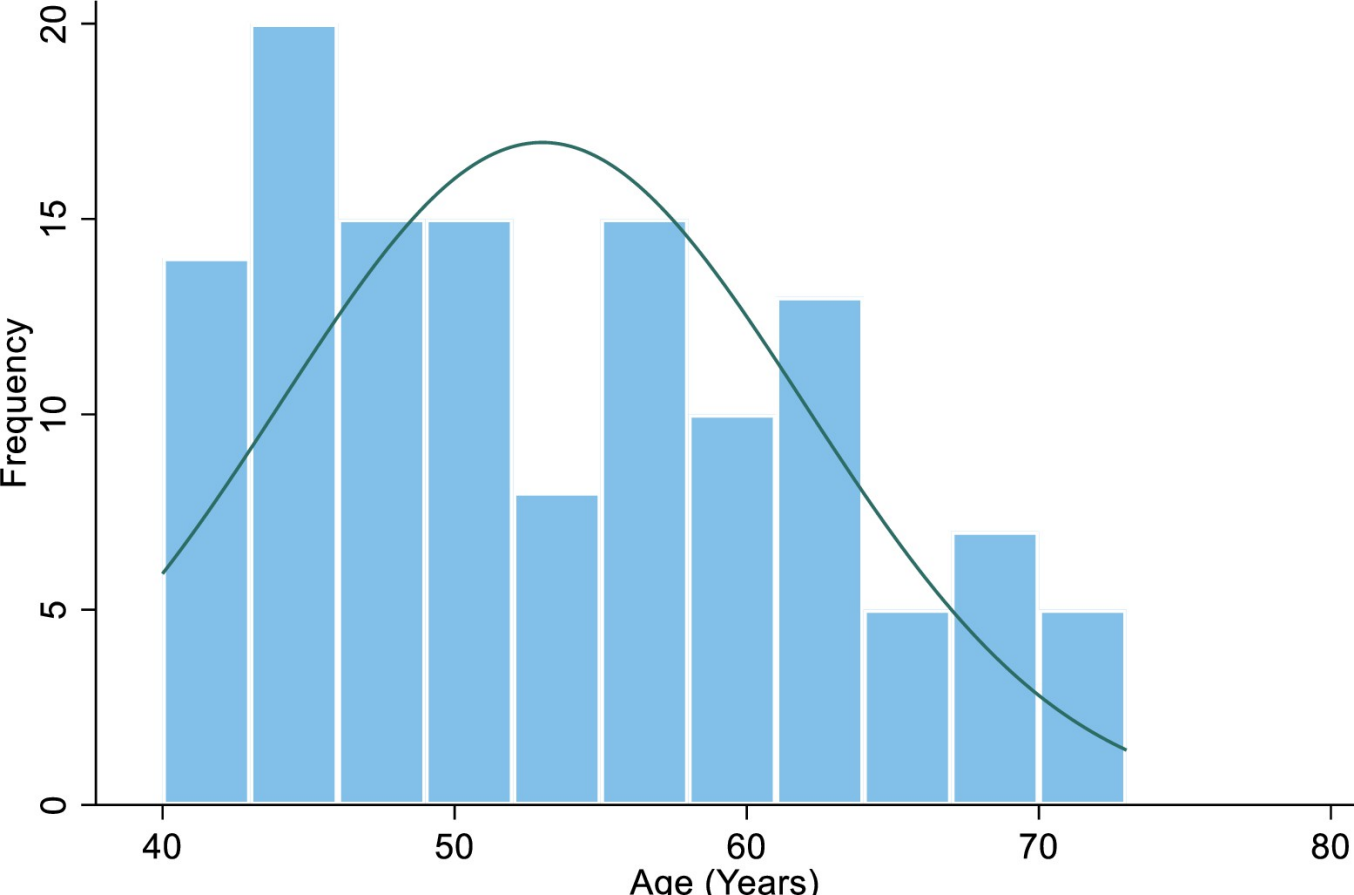
Age distribution of subjects.

Among 235 eligible participants, 228 consented and participated in the study (participation rate: 97.0%) Fig 1. Reasons for non consent to participation were, research not supported by the participant’s religious belief, research is of no benefit to the participant, lack of trust regarding use of research information, and request for monetary compensation before accepting to participate. 119[52.2%] participants were female, 109[47.8%] were male with a F:M = 1.1 The median age of our study participants was 64 years with an interquartile range of 50–76 years. One hundred and eighteen (51.8%) participants had secondary or tertiary education, 117(51.3%) participants were either unemployed or retired. 70 (30.7%) participants had a first degree relative with a diagnosis of glaucoma. Fig 2 is a histogram showing that the age of participants does not follow a normal distribution, while Table 1 shows the socio-demographic profiles of participants.

**Table 1:** Socio-demographic characteristics.

Variables	Frequency(n)	Percentage(%)	Median(IQR)
**Age (Years)**			64(50–76)
41–60	174	76.4	
60–90	54	23.6	
Total	228	100.0	
**Gender**			
Female	119	52.2	
Male	109	47.8	
Total	228	100.0	
**Marital Status**			
Married	203	89.0	
Single	7	3.1	
Widowed	18	7.9	
Total	228	100.0	
**Education**			
No response	7	3.1	
No education	29	12.7	
Primary	74	32.5	
Secondary	46	20.2	
Tertiary	72	31.6	
First degree	68	94.4	
Masters	2	2.8	
PhD	2	2.8	
**Total**	228	100.0	
**Employment**			
No response	3	1.30	
Artisan	2	0.90	
Business man	14	6.10	
Civil servant	25	11.10	
Farming	4	1.80	
Professional	14	6.10	
Retired	46	20.20	
Self employed	46	20.20	
Student	1	0.43	
Teacher	1	0.43	
Unemployed	71	31.10	
Total	228	100.0	

IQR

٭ = Interquartile Range

### Willingness, motivation to participate in genetic research and associations

Among 219 [96.0%] participants who expressed willingness to participate in glaucoma genetic research, 213(93.8%) also expressed willingness to submit blood samples for the study, however, the willingness to participate is significantly reduced to 27(11%); Pearson’s Chi Square (χ^2^ = 18.59, P<0.001), if there will be no feedback of the results of the genetic study to the study participants. 193(84.6%) of them will allow their spouse or child to participate in glaucoma genetic research. Among these, 33.2% have a first degree relative with a diagnosis of glaucoma. Eight (3.5%) participants only, expressed willingness to participate in glaucoma genetic research if the study would require submission of their body samples after death, whereas no participant (0%) would be willing to have their ocular tissue sampled for glaucoma genetic research after death. Table 2 shows willingness to participate profiles and motivating factors for subject participation in glaucoma genetic research. The most common reason for participating in glaucoma genetic research among our study participants who are willing to participate was the desire to obtain more knowledge about the disease in 143 (62.9%) participants. Other motivating factors include desire to obtain medical treatment for my disease in 27(11.8%) and to help future generation in 19(8.3%). 15(6.6%) are motivated by the desire to contribute to knowledge, while 5(2.2) are motivated to participate in genetic research so as to obtain a cure for glaucoma.

**Table 2:** Willingness and motivation to participate profiles among subjects.

Variable	Yes(% Freq.)	No(% Freq.)	Total(n)
**Willingness to participate**			
Will you be willing to participate in glaucoma genetic research?	219(96.0)	9(4.0)	228
Willingness to allow spouse/child participate in glaucoma genomic research?	193(84.6)	35(15.4)	228
Will you accept to submit a blood sample for glaucoma genetic research if invited to do so?	213(93.8)	14(6.2)	227
Will you be willing to submit body sample for glaucoma genomic research after death?	8(3.5)	220(96.5)	228
Will you be willing to donate your eyes or ocular sample for glaucoma genomic research after death	0(0)	228(100)	228
*Willingness to participate in genomic research if your personal result will not be given you	27(11.9)	200(88.1)	227
** Motivation for participation**	**n(% freq.)**		**Total**
To gain more knowledge about glaucoma	143(62.9)		228
To obtain medical treatment for my disease	27(11.8)		228
I want to help future generation	19(8.3)		228
To honor the invitation	17(7.5)		225
I want to contribute to knowledge	15(6.6)		227
I want to help find a cure for glaucoma	5(2.2)		228
To obtain free drugs	0(0)		225

*Pearson’s Chi Square (χ^2^ = 18.59, P<0.001)

Table 3 evaluates the association between socio-demographic factors and the dependent variable willingness to participate in glaucoma genetic research using Pearson’s chi square test. Only age category (χ^2^ = 6.17, degrees of freedom = 1) P<0.05 was significantly associated with willingness to participate in glaucoma genetic studies among our study participants.

**Table 3:** Test of association between the willingness to participate and Socio-demographics using the chi square test.

Variables	Willingness to participate in Glaucoma genetic research				P-values
	No		Yes		
	Freq.	%	Freq.	%	
**Age category (Years)**					*0.013
41–60	80	88.9	95	68.8	
61–90	10	11.1	43	31.2	
Total	90	100.0	138	100.0	
**Gender**					0.720
Female	62	77.5	125	84.4	
Male	18	22.5	23	15.6	
Total	80	100.0	148	100.0	
**Marital Status**					0.241
Married	85	95.5	124	89.1	
Single	3	3.4	3	2.3	
Widowed	1	1.1	12	8.6	
Total	89	100.0	139	100.0	
**Education**					0.413
None	1	7.2	28	13.6	
Primary	5	35.7	69	33.5	
Secondary	3	21,4	42	20.4	
Tertiary	5	35.7	67	32.5	
Total	14	100.0	206	100.0	
**Employment**					0.974
No Response	0	0.0	7	3.3	
Artisan	0	0.0	2	0.9	
Businessman	0	0.0	14	6.5	
Civil servant	2	14.3	23	10.8	
Farming	0	0.0	4	1.9	
Professional	1	7.1	12	5.6	
Retired	2	7.1	43	20.0	
Self employed	5	35.7	41	19.2	
Student	0	0.0	1	0.5	
Teacher	0	0.0	1	0.5	
Unemployed	5	35.7	66	30.8	
Total	14	100.0	214	100.0	
Family History of Glaucoma					
Yes	5	86.7	197	97.0 **	
No	1	13,3	6	3.0	
Total	6	100.0	203	100.0	

* *Pearson’s* Chi Square (χ^2^ = 6.17, degrees of freedom = 1) P<0.05

** Fishers Exact = 0.15, P = 0.86

### Determinants of willingness to participate using logistic regression

Crude (bivariate) analysis (Table 4) showed that age (61–90) years was associated with willingness to participate in genomic research (OR = 3.3, P = 0.011) and willingness to submit blood samples (OR = 3.0, P = 0.015) Table 3 shows results of a crude analysis performed with bivariate logistic regression. After adjusting for other covariates using multivariable logistic regression, our study found that glaucoma patients who were aged between 61 years and 90 years (P = 0.004, OR = 7.2) were 7 times more likely to express willingness to participate in glaucoma genetic research. There was no significant association between willingness to participate in glaucoma genetic research and other covariates including gender (P = 0.44), marital status(P = 0.51), educational status(P = 0.22), family history of glaucoma (P = 0.26) and employment (P = 0.68) in our adjusted analysis. There was no effect modification by other covariates in the relationship between willingness to participate in genetic research and age. We tested for the likelihood of effect modification by generating interaction terms between age category and other covariates in our multivariate model in Table 5. However, the P values obtained for our interaction terms were not statistically significant: agecat*gender P = 0.567, agecat*education P = 0.908, agecat*familyhxglaucoma P = 0.318, agecat*maritalstatus P = 0.737.

**Table 4:** Crude (Unadjusted) analysis evaluating the relationship between willingness to participate in genetic research and covariates using binary logistic regression.

Independent variables	B	S.E.	OR	95% C.I. for OR		P-values
				Lower	Upper	
Age category	1.201	0.499	3.321	1.247	8..847	*0.016
Gender	0.303	0.473	1.354	0.536	3.422	0.529
Marital Status	-2.422	1.559	0.089	0.004	1.885	0.120
Education	-0.052	0.185	0.949	0.659	1.365	0.778
Employment	-0.048	0.775	0.953	0.819	1.109	0.536
Family history of glaucoma	-0.238	0.377	0.788	0.376	1.651	0.528

*Age category significantly predicted willingness to participate in a genetic study in this bivariate model.

**Table 5:** Multivariate (Adjusted) analysis evaluating association between willingness to participate in a genetic research and covariates.

Independent variables	B	S.E.	OR	95% C.I. for OR		P-values
				Lower	Upper	
Age category	1.986	0.681	7.291	1.917	27.699	*0.004
Gender	0.421	0.544	1.527	0.524	4.428	0.439
Marital Status	-0.604	0.909	0.546	0.091	3.246	0.506
Education	0.351	0.285	1.421	0.813	2.484	0.217
Employment	-0.041	0.098	0.960	0.792	1.163	0.676
Family History of glaucoma	-0.543	0.478	0.599	0.228	1.483	0.256
Constant	-0.603	1.737	3.91	0.035	436.823	0.570

*Age category significantly predicted willingness to participate in a genetic study in this multivariate model.

We therefore infer that among our study participants, the effect of age on willingness to participate in glaucoma genetic research does not vary with the levels of other study covariates.

Both crude(bivariate) analysis, (p = 0.023) and adjusted (multivariate) analysis, p = 0.01 showed that our study participants with family history of glaucoma would be more willing to allow their children or spouse to participate in a glaucoma genetic study. The adjusted analysis shows that accounting for age, gender, education, employment and marital status, family history of glaucoma is significantly associated with the willingness of a person with glaucoma to allow his child or spouse participate in a glaucoma genetic study (Table 6).

**Table 6:** Relationship between willingness to allow child or spouse to participate in a glaucoma genetic study and family history of glaucoma.

Variable	Coefficient	p-value	[95% conf. Interval]
A. Univariate Model(Crude Analysis)			
Family History of Glaucoma	0.072674	0.023	0.01009 0.13525
_Cons	0.952992	0.000	0.83459 1.07139
B. Multivariate Analysis			
Family History of Glaucoma	0.079457	0.018	0.01351 0.14539
Age	-0.0005699	0.785	-.0046836 .0035438
Gender	-.0510778	0.240	-.1365856 .034437
Education	-.0195212	0.326	-.0586688 .0196265
Employment	-.0157479	0.575	-.070539 .0355471
Marital Status	-.0076388	0.837	-.0807539 .0654763
Constant	1.126261	0.000	.7419989 1.510522

## Discussion

### Determinants of willingness to participate in glaucoma genetic research

In our index study, the willingness to participate in glaucoma genomic research and submit blood samples among glaucoma participants were high at 96% and 93.9% respectively. The high expression of willingness for participation in glaucoma genomic research is further highlighted by the finding that four of five participants will encourage their relatives to participate as well. As more glaucoma genetic studies are designed and conducted in the foreseeable future in our setting, our findings perhaps may represent a salient promissory note for a high subject enrolment. This finding is in contrast to a report by Parikh et al., in which only 38.6% [190/492] of African American participants were willing to enroll in a glaucoma genetic research [22]. However their study included both glaucoma patients as well as controls [22] whereas our index study included only glaucoma patients. Many reasons have been suggested as contributory to the low participation of African Americans in genetic studies, this includes the effect of the well reported ethical abuses and distrust arising from previous studies on African Americans such as the Tuskegee Syphilis Study which do not directly affect participants of our study [23, 24]. However this observation could be changing because in a study by Scot et al., among persons of African descent, they found a high willingness (87%) to participate in genomic research related to cancer, diabetes and other chronic diseases [25]. The observation that glaucoma patients from our setting may perhaps harbor a higher willingness to participate in glaucoma genetic studies highlights the need for further studies to elucidate these findings. However, the encouraging optimism generated by such a high willingness profile is tempered by the markedly reduced willingness to participate if the results of such studies are not fed back to subjects.

Feedback of genetic research to participants is an evolving subject characterized by divided opinion in contemporary scientific discourse. From a previous study by Sanderson and coworkers in a minority African American and Hispanic population in the United States, there was considerable willingness to participate in genomic research as well as a desire for feedback of personal results [21]. This view was also supported by Budin-Ljøsne et. al. who suggested that results of genetic studies that are “scientifically robust, analytically valid, and clinically actionable should be offered to research participants” [26]. However, other researchers argue that such feedback may break ethical codes that protect subjects’ identities and potentially lead to harm with loss of autonomy [27]. They therefore suggested the need for an intermediary “key holder” to control access to patients database [27]. Another major counter argument to feedback of genetic research in general, concerns the fundamental distinction between research and treatment which presumes that unlike in treatment where physicians are bound by an ethical obligation to communicate results to individual patients, researchers are under no such obligations [28]. Whereas profound discussions are currently championed by researchers from Europe and North America, Africa has remained largely underrepresented although the continent is reported to possess the greatest genetic diversity in comparison to other populations worldwide [28, 29]. This study was conceived to provide the first scientific evidence from an African context on the normative determinants of participation in genetic studies using the thematic window of glaucoma research. From our study, willingness to participate in glaucoma genomic research is significantly higher with the promise of result feedback (χ^2^ = 18.59, P<0.001). Possible explanations for this finding could be the desire among participants to obtain more information about glaucoma including possible causal factors and outcomes so as to plan for the future as well as an expectation of reciprocity from researchers for using their biological samples in a study. In a study by Ralefala and co-workers, awareness, improving lifestyle, accepting ones situation and preparing for the future were identified as reasons for participants preference for feedback of genomic research results [20]. Feedback could be in the form of general research results or personal research results. It is our considered opinion that feedback of results should be prioritized in the conception, design and implementation of glaucoma genetic research. A contemplation of result feedback should involve an ethically efficient mechanism that is clinically meaningful as well as contextually responsive and feasible.

### Socio-demographic profile, willingness to participate and related factors

The socio-demographic profile of participants shows an almost balanced gender ratio (F:M = 1.1). The median age of 64 years shows that most participants were in their late middle age and over half of them were either retired or unemployed. The socio-demographic profile of our study participants is different from those of previously published series on glaucoma from our sub region [5, 30]. While the predominant age group was 60 years and below in our study, other studies found most participants at 60 years of age and above [5, 30]. These disparities may have partly resulted from the remarkable differences between the highly conservative enrolment criteria of our study and those of the previously cited studies [5, 30]. We found the elderly age group (61–90 years) to be significantly associated with a high willingness to participate in glaucoma genetic research OR = 7.2, when compared to younger subjects aged 41-60years. Although this finding provides perhaps a salient demographic capital for researchers planning a glaucoma genetic research in our setting, we do not know the reason for this age related disparity in willingness profiles. We are however aware that old age and African ancestry are notable risk factors for glaucoma [11], hence a high willingness to participate in glaucoma genetic research by a high risk population is welcoming in this regard.

In addition, the higher willingness to participate among older glaucoma patients who may have harbored the disease for a longer period and therefore experienced a more protracted duration of suffering from the progressive visual disability associated with glaucoma may be altruistic. In that case, their motivation could be explained by the incentive that glaucoma genetic research may represent a salient path in the search for glaucoma cure. However, our findings on the relationship between age and willingness to participate in genomic research differs from the published findings from a genomic study conducted among participants with multiple sclerosis by Cucarro and co workers which showed that younger patients were more likely to participate in the genetic study for multiple sclerosis when the motivation was to find a cure for multiple sclerosis compared to older patients [31]. The impact of physical disability associated with multiple sclerosis for the younger active population may account for this difference in addition to the fact that they are still early in the disease process.

Although other covariates did not significantly influence the profile of willingness to participate, the result of our crude and adjusted analysis may suggest that gender, education, family history and employment may likely confound the effect of age on the willingness to participate in genetic research. However, they did not significantly influence willingness of participants in our index study. A previously reported study on factors associated with participation by African Americans in glaucoma genetic research also showed that demographic factors such as gender, education and socioeconomic status did not influence enrolment [22]. In addition a study by Georgina and coworkers on glaucoma genetic risk testing, reported that age, gender, level of education did not influence interest in glaucoma genetic risk testing [32].

The level of education did not influence the willingness to participate in the research. It is possible that exposure to adequate basic information on genomic research using the patient information sheet prior to administration of our study questionnaire may have nullified the effect of educational status on the willingness to participate in the genomic study since all participants irrespective of educational status received basic information about glaucoma genetic research. This is collaborated by Rahim and coworkers in their study which reported that basic literacy in genetics was associated with willingness to participate in research [33].

Our study shows that 193(84.6%) participants would be willing to allow their spouse or child to participate in a glaucoma genomic research. In addition, study participants with family history of glaucoma would be more willing to allow their children or spouse to participate in a glaucoma genetic study. This finding is important for the following reasons. First, as glaucoma is genetically transferable from parents to their off springs, participation of children of glaucoma patients in genetic research will help in early diagnosis and treatment with possible improved visual outcomes. Further participation of children of patients with glaucoma could provide more information and useful insights on the possible determinants as well as salient patterns of glaucoma inheritance and transmission among family progenies. Therefore, persons with a family history of glaucoma are a veritable source for the recruitment of family members in glaucoma genetic research compared to those without a family member with glaucoma.

### Marital status did not influence willingness to participate in glaucoma genetic research

It is likely that in our study setting, decision-making regarding participation in glaucoma genomic research may not be influenced by a spouse. This finding may reflect the degree of autonomy study participants exhibit regarding their participation in glaucoma genetic research. However, Marshall and coworkers in their genetic study on breast cancer which was carried out in southwest Nigeria reported that a fifth of the married participants would seek permission from their husbands before participating in the genetic research [34].

From our study, we believe that strategies aimed at improving participation in glaucoma genomic research in our setting should prioritize the age of participants over educational, employment status and gender. Family history of glaucoma should be considered when recruiting relatives of glaucoma patients.

Type of biologic specimen was another consideration for participation in glaucoma genetic research. Biological samples for glaucoma genomic research include blood samples, ocular tissues removed during surgery and ocular tissue donated after death. In our study, majority of the participants would submit blood samples for research while none of the participants would submit ocular tissue for research after death. However, in contrast a study from the United states reported that 90% of ophthalmic patients were willing to donate eyes as well as join the eye donation registry although only 12% would do so specifically for research purposes [35]. From this US report, African Americans were less likely to donate eyes compared with Caucasians. In a German study, Murist et al found that 77% participants would agree to donate their eyes for research [36]. These reports may perhaps highlight the likelihood of a racial bias in the willingness to donate ocular specimens for research after death. We are of the opinion that an interplay of contextual factors may be responsible for the current low willingness for ocular specimen donation among Africans and related races.

The contextual factors which may account for the intriguing refusal by 96% of our study participants may include strong religious and customary beliefs and practices that are common in our study setting. Among such customary beliefs is reincarnation which teaches that dead humans incarnate again and return to life in their physical bodies at a future time after death [37]. It is also widely believed that an intact human body at the time of burial is a necessity for a wholesome reincarnation [38]. Therefore donation of an eye may render the person “incomplete” and hence may result to anophthalmos in the next incarnation.

The strong instrumental attitudes that motivated participation in glaucoma genetic research were mainly to gain more knowledge of the disease, obtain medical treatment, contribute to knowledge and help future generations. From a recently published study on African American cohort which applied an Integrated behavioral model to assess recruitment strategies in glaucoma genetic research, identified self-reported motivations for participating in this care-focused and community-promoted research program included learning more about personal health and contributing to future care options for others as well [39].

The varied scientific quest to unravel the causal linkage between some identified genetic mutations and subsets of primary open angle glaucoma is driven by the speculation that these research forays may yield dividends by opening new channels including new signaling pathways, biomarkers, epigenetic factors and further genetic studies that may converge at a final common path for establishing causality [40]. These studies in the glaucoma research pipeline may also lead to new drug discoveries and treatment modalities [1, 40–42]. It is therefore significant that the rich genetic capital of Africa should serve as a contextually valuable resource in the journey to resolve glaucoma causality. We believe that all current efforts invested in glaucoma research are justified considering the fact that glaucoma represents the most common cause of irreversible blindness [6]. We believe that understanding the determinants of participation in genetic research as well as associated instrumental motivating factors will help to maximize the investment of Africa’s deep and diverse ancestral endowments in glaucoma research [43].

## Conclusion

Willingness to participate in glaucoma genetic research is higher among the elderly and participant enrolment is likely to be higher if participants will receive result feedback.

### Limitations

We did not evaluate the contextual options for feedback of genetic results as well as time varying trends in participants’ responses.

## Supporting information

S1 File (DOCX)

S2 File (XLSX)

S1 Questionnaire (DOCX)
