# Supplementary material for: Determinants of participation in glaucoma genomic research in South East Nigeria: A cross-sectional analytical study
Source: PLoS One. 2023 Nov 17;18(11):e0289643. doi: 10.1371/journal.pone.0289643 (PMC10655997; doi:10.1371/journal.pone.0289643)
Supplement: S1 File — (DOCX) [file pone.0289643.s001.docx]

**Participant information sheet**

*The strategy to explain genetics and genomics to research participants will be derived from strategies used by researchers in the H3Africa research project.*

Explaining about genomic research:

Heredity: DNA is a code that you inherit from your parents and that you pass on to your children. This information may also be passed on from parent to child. This kind of information is passed from the father and the mother to their children and on to their grandchildren in other words from one generation to the next.

Heredity and health: some illnesses are passed down in families because our DNA comes from our parents. Also, some inherited traits give a person a health advantage, people with that change will be more likely to survive and pass the change unto their families.

Genes and disease causation: some sicknesses can be caused by problems with DNA. Studying genes along with health information will help the researcher better understand what causes diseases.

Heredity and phenotype: these genes are present in all of us and are what make people in families look like each other, but different from others. For example, some families are taller or shorter than others. Some families may have a recurring type of sickness seen in many generations.

A part of the body can be tested to determine if a person has a particular inherited disease, or is at risk of a particular disease, or is likely to develop a particular disease in future

Blood samples can be used to check the genes that make up a person

It is possible that while checking for a gene that causes glaucoma, some other genes that cause another disease can be discovered.

It is possible that health information about your family members can be discovered by testing your blood sample

It is possible that your medical information and blood sample will be shared with others researchers in other countries
