# Supplementary material for: Determinants of participation in glaucoma genomic research in South East Nigeria: A cross-sectional analytical study
Source: PLoS One. 2023 Nov 17;18(11):e0289643. doi: 10.1371/journal.pone.0289643 (PMC10655997; doi:10.1371/journal.pone.0289643)
Supplement: S1 Questionnaire — (DOCX) [file pone.0289643.s003.docx]

Questionnaire

**SECTION 1**

**1. Demographics**

a. Identification number………..

b. Age at last birthday …………...

c. Gender 1. male 2. female

d. Marital status 1. single 2. married 3. separated/divorced 4. widowed

e. Educational qualification 1. none 2. primary 3. secondary 4. university 5. others……….

f. Employment status 1. Professional 2. self employed 3. unemployed 4. retired 5. student 6. housewife 7. others

h. Do you have glaucoma/are you being treated for glaucoma 1. yes 2. no 3. don’t know

i. Does any member of your family have glaucoma. (Ist degree relatives) 1. yes 2. no 3. don’t know If Yes, mention who: brother, sister, mother, father, children ,

2a**. Awareness and Knowledge**

a. Have you heard about glaucoma………………………………………………………………. 1.yes 2. No

b. Glaucoma is (tick) 1. an eye infection that causes visual loss 1.yes 2.No

2. an eye disease that can be transferred through contact 1. yes 2. No

3. an eye disease that is caused by high eye pressure 1. yes 2. No

4. caused by evil spirits 1.yes 2. no

5. can cause blindness which is not reversible 1.yes 2. no

6. blindness from glaucoma is reversible 1. yes 2. No

7. common among blacks 1. yes 2. no

8. an inherited disease 1. yes 2. no

9. an eye disease that occurs due to high blood pressure 1.yes 2. No

10. an eye disease that occurs due to diabetes 1. yes 2. No

3.a Have you participated in a research where body samples where taken for a condition where disease is passed from one generation to another generation? 1. yes 2. no 3. don’t know

If yes was it a genetic/genomic research (genomic research is a type of study to determine if a disease can be passed from a person to another through a code known as genes) . 1. yes 2. No 3. don’t know

4. **Willingness to participate in glaucoma genomic research**

a. i. Do you know about genetic/genomic research?..............................................................

ii. Have you heard about genetic/genomic research?...........................................................

iii. What do you understand by genetic/genomic research?..................................................

iv. Can you tell me what you mean if you say that a disease/ behavior/ability is genetic………………………………………………………………………………………………………………………………………………

………………………………………………………………………………………………………………………………………………………….

v. Where do you think genes are located in the body ……………………………………………………………………..

vi. Is glaucoma a genetic disease? …………………………………………………………………………………………………..

vii. Is glaucoma an inherited disease? ……………………………………………………………………………………………..

( use the patient information sheet to explain what genomic research is and its associated risks and benefits using simple language)

b. are you willing to participate in a glaucoma genetic research?, a research that explores how glaucoma is transferred from one generation to another through genes. 1. yes 2. no

c. if you were invited to participate in a research were you would submit a blood sample to check if you have the glaucoma gene , will you participate?........................................................................................

d. will you be willing to allow your spouse participate in the glaucoma genomic research 1. yes 2. no

g. will you be willing to submit a your body sample for genetic/genomics research after death for a study involving other eye diseases 1. yes 2. No

h. will you be willing to donate your eyes for genetic/genomic research after death 1.yes 2.no

i. are you willing to participate in a genomic research if your personal research result will not be given to you…………………………………………

will you participate in a genomic research only if your personal research results will be returned to you ………………………………………………………

h. What are your reasons for participating in glaucoma genetic research?

1. I want to know more about my illness

2. I want to get medical care for my illness

3. I was invited to participate

4. I will be given some drugs

5. I want to please the research team

6. I want to help contribute to scientific knowledge

7. I want to help future generations

8/ I want to help find a cure for glaucoma

8. others: ……………………………………………

i. What are your reasons for not participating in glaucoma genetic research [ANSWER THIS QUESTION IF RESPONSE TO QUESTION 4b IS NO]

1. I do not think it will benefit me personally

I do not trust what will happen to my sample

I do not have time

I do not want to participate for religious reasons

**SECTION 2**

**Deciding to participate**

1. would you need to obtain permission from someone else before participating in genomic research 1. yes 2. no

2. if yes who would you consult?.........................................................

3. would you participate in genomic research if you were offered free eye tests, free eye drops? 1. yes 2. no

4. how long will it take you to decide to participate in a genomic research? …………………………………………….

**Views about donating DNA via blood sample**

1. Would you allow information derived from your blood sample to be preserved for future use

( 1. yes 2. no 3. don’t know)

1. Would you let your blood sample to be shipped to another country for further research
2. Would you let your blood sample to be used for another research during storage of samples
3. Would you let your blood sample to be preserved for future unspecified use
4. Would you let your blood sample and medical information to be shared with i) non profit researchers ii) for profit researchers [that is researchers who can make money through result from your blood sample]
5. Would you let your blood sample to be used for another research different from this present research
6. Would you let your blood sample to be used in only this present research
7. Would you want to be recontacted if information derived from your blood sample will be used in another research
8. Would you want to be recontacted if information derived from your blood sample will be sent to another researcher

**Benefit and harm from participating in research**

From the benefits of research shared with you which one is of most important to you……………………………………………………………………………..

From the risks of research shared with you which one is of most concern to you ………………………………………………………………………………………………………..

d. There is a possibility that all your genetic information could be accessed by the researchers including genetic health risks, do you have any concerns about this…………………………………………………

**Views about return of results and incidental findings**

1. Do you expect to know the findings from the blood sample that will be taken from you? 1. Yes 2. no

2. Do you expect your personal results to be returned to you? 1. Yes 2. no

3. Do you expect to receive a general result on the research that will be done with your blood sample and that of other persons 1. Yes 2. no

4. Do you expect to receive results on other findings that maybe discovered with your blood? 1 yes 2 no

5. It is possible that other diseases maybe discovered in your blood sample, do you expect to be notified if other diseases are discovered? 1. Yes 2. no

6. Which type of result do you expect to be returned to you?

A. results about the entire research

B. your personal result about glaucoma

C. Result about other diseases that maybe discovered

D. All of the above

**It is possible to discover other diseases apart from glaucoma**

a. would you want to receive a result if another disease is discovered which cannot be treated or prevented 1. yes 2. no

b. would you want to receive a result if another disease is discovered which can be treated or discovered 1. yes 2. no

c. Do you want to receive results about other diseases that may be discovered? 1. yes 2. No

How would you want to receive your personal result?.....................................................................

a. Via a phone call b. via an email c. through my eye doctor d. through the media e. others……………………………………………………………………………………..

**Views on withdrawal of blood sample already submitted for research**

a. would you for any reason wish to withdraw your blood sample from research 1. yes 2. no

b. why would you want to withdraw your blood sample:………………………………………………………….
